# Multisensory and sensorimotor interactions in speech perception

**DOI:** 10.3389/fpsyg.2015.00458

**Published:** 2015-04-20

**Authors:** Kaisa Tiippana, Riikka Möttönen, Jean-Luc Schwartz

**Affiliations:** ^1^Institute of Behavioural Sciences, University of HelsinkiHelsinki, Finland; ^2^Department of Experimental Psychology, University of OxfordOxford, UK; ^3^Grenoble Images Parole Signal Automatique-Lab, Speech and Cognition Department, Centre National de la Recherche Scientifique, Grenoble UniversityGrenoble, France

**Keywords:** audiovisual, cognitive disorders, learning, McGurk effect, multisensory, sensorimotor, somatosensory, speech perception

This research topic presents speech as a natural, well-learned, multisensory communication signal, processed by multiple mechanisms. Reflecting the general status of the field, most articles focus on audiovisual speech perception and many utilize the McGurk effect, which arises when discrepant visual and auditory speech stimuli are presented (McGurk and MacDonald, 1976). Tiippana (2014) argues that the McGurk effect can be used as a proxy for multisensory integration provided it is not interpreted too narrowly.

Several articles shed new light on audiovisual speech perception in special populations. It is known that individuals with autism spectrum disorder (ASD, e.g., Saalasti et al., 2012) or language impairment (e.g., Meronen et al., 2013) are generally less influenced by the talking face than peers with typical development. Here Stevenson et al. (2014) propose that a deficit in multisensory integration could be a marker of ASD, and a component of the associated deficit in communication. However, three studies suggest that integration is not deficient in some communication disorders. Irwin and Brancazio (2014) show that children with ASD looked less at the mouth region, resulting in poorer visual speech perception and consequently weaker visual influence. Leybaert et al. (2014) report that children with specific language impairment recognized visual and auditory speech less accurately than their controls, affecting audiovisual speech perception, while audiovisual integration *per se* seemed unimpaired. In a similar vein, adult patients with aphasia showed unisensory deficits but still integrated audiovisual speech information (Andersen and Starrfelt, 2015).

Multisensory information can influence response accuracy and processing speed (e.g., Molholm et al., 2002; Klucharev et al., 2003). Scarbel et al. (2014) show that oral responses to speech in noise were faster but less accurate than manual responses, suggesting that oral responses are planned at an earlier stage than manual responses. Sekiyama et al. (2014) show that older adults were more influenced by visual speech than younger adults and correlated this fact to their slower reaction times to auditory stimuli. Altieri and Hudock (2014) report variation in reaction time and accuracy benefits for audiovisual speech in hearing-impaired observers, emphasizing the importance of individual differences in integration. Finally, Heald and Nusbaum (2014) show that when there were two possible talkers instead of just one, audiovisual information appeared to distract the observer from the task of word recognition and slowed down their performance. This finding demonstrates that multisensory stimulation does not always facilitate performance.

While multisensory stimulation is thought to be beneficial for learning (Shams and Seitz, 2008), evidence for this is still scarce. In the current research topic, the overall utility of multisensory learning is brought under question. In a paradigm training to associate novel words and pictures, Bernstein et al. (2014) show no benefit of audiovisual presentation compared with auditory presentation for normal hearing individuals, and even a degradation for adults with hearing impairment. In a study of cued speech, i.e., specific hand-signs for different speech sounds, Bayard et al. (2014) demonstrate that individuals with hearing impairment used the visual cues differently from their controls, even though both groups were experts in cued speech. Kelly et al. (2014) show that when normal hearing adults learned words in a foreign language, viewing or producing hand gestures accompanying audiovisual speech did not affect the outcome. Lee and Noppeney (2014) show that musicians had a narrower audiovisual temporal integration window for music, and to a smaller extent also for speech, implying that the effect transfers from the practiced music stimuli also to other stimulus types. Together, these findings suggest that long-term training and active use may be requisites for multisensory information to be useful in learning speech.

Neurophysiological correlates of audiovisual speech perception were addressed in the research topic. By using electroencephalography (EEG) it was shown that attention (Alsius et al., 2014) and stimulus context (Ganesh et al., 2014) affected early event-related potentials (ERPs) to audiovisual speech. This provides further evidence that audiovisual interactions are not completely automatic. By using functional magnetic resonance imaging, Erickson et al. (2014) demonstrate a subdivision of posterior superior temporal areas for integrating congruent vs. incongruent audiovisual speech, and Callan et al. (2014) show that different regions in the premotor cortex were involved in unisensory-to-articulatory mapping and audiovisual integration.

Interactions between auditory and motor brain areas during auditory speech perception were also investigated. By using magnetoencephalography, Alho et al. (2014) demonstrate that connectivity between auditory and motor areas increased from passive listening to clear speech to listening to speech in noise, and that the strength of this connectivity was positively correlated with the accuracy of syllable identification. Moreover, analyses of EEG oscillations revealed that alpha and beta rhythms generated in the sensorimotor and auditory areas were modulated during syllable discrimination tasks (Bowers et al., 2014; Jenson et al., 2014). By using theta-burst transcranial magnetic stimulation, Rogers et al. (2014) show that disrupting the lip area of the motor cortex impaired discrimination of lip-articulated speech sounds from sounds not articulated on the lips. The involvement of the motor processes is often considered to make speech perception “special,” i.e., essentially different from perception of non-speech stimuli. However, this remains a highly controversial view. Carbonell and Lotto (2014) claim that speech should not be considered special amongst other stimuli with regards to multisensory integration.

Somatosensory information can also influence speech perception. Ito et al. (2014) used EEG to study how stretching the skin on both sides of the mouth influences processing of speech sounds, and displayed auditory-somatosensory interaction that was sensitive to intersensory timing. In another EEG study, Treille et al. (2014) report that haptic exploration of the talker's face during speech perception modulated ERPs. These findings confirm that auditory-somatosensory interactions contribute to speech processing.

The current research topic shows that speech can be perceived via multiple senses and that speech perception relies on sophisticated unisensory, multisensory and sensorimotor mechanisms. Multisensory information can facilitate perception and learning of speech. Still, there is great variation in multisensory perception and integration in both typical and special populations at different ages, which should be studied further in the future.

## Conflict of interest statement

The authors declare that the research was conducted in the absence of any commercial or financial relationships that could be construed as a potential conflict of interest.

## References

[B1] Alho J. Lin F. H. Sato M. Tiitinen H. Sams M. Jääskeläinen I. P. (2014). Enhanced neural synchrony between left auditory and premotor cortex is associated with successful phonetic categorization. Front. Psychol. 5:394. 10.3389/fpsyg.2014.00394 24834062 PMC4018533

[B2] Alsius A. Möttönen R. Sams M. E. Soto-Faraco S. Tiippana K. (2014). Effect of attentional load on audiovisual speech perception: evidence from ERPs. Front. Psychol. 5:727. 10.3389/fpsyg.2014.00727 25076922 PMC4097954

[B3] Altieri N. Hudock D. (2014). Hearing impairment and audiovisual speech integration ability: a case study report. Front. Psychol. 5:678. 10.3389/fpsyg.2014.00678 25071649 PMC4076931

[B4] Andersen T. S. Starrfelt R. (2015). Audiovisual integration of speech in a patient with Broca's Aphasia. Front. Psychol. 6:435 10.3389/fpsyg.2015.00435 PMC4411977 25972819

[B5] Bayard C. Colin C. Leybaert J. (2014). How is the McGurk effect modulated by Cued Speech in deaf and hearing adults? Front. Psychol. 5:416. 10.3389/fpsyg.2014.00416 24904451 PMC4032946

[B6] Bernstein L. E. Eberhardt S. P. Auer E. T. (2014). Audiovisual spoken word training can promote or impede auditory-only perceptual learning: results from prelingually deafened adults with late-acquired cochlear implants and normal-hearing adults. Front. Psychol. 5:934. 10.3389/fpsyg.2014.00934 25206344 PMC4144091

[B7] Bowers A. L. Saltuklaroglu T. Harkrider A. Wilson M. Toner M. A. (2014). Dynamic modulation of shared sensory and motor cortical rhythms mediates speech and non-speech discrimination performance. Front. Psychol. 5:366. 10.3389/fpsyg.2014.00366 24847290 PMC4019855

[B8] Callan D. E. Jones J. A. Callan A. (2014). Multisensory and modality specific processing of visual speech in different regions of the premotor cortex. Front. Psychol. 5:389. 10.3389/fpsyg.2014.00389 24860526 PMC4017150

[B9] Carbonell K. M. Lotto A. J. (2014). Speech is not special… again. Front. Psychol. 5:427. 10.3389/fpsyg.2014.00427 24917830 PMC4042079

[B10] Erickson L. C. Zielinski B. A. Zielinski J. E. Liu G. Turkeltaub P. E. Leaver A. M.. (2014). Distinct cortical locations for integration of audiovisual speech and the McGurk effect. Front. Psychol. 5:534. 10.3389/fpsyg.2014.00534 24917840 PMC4040936

[B11] Ganesh A. C. Berthommier F. Vilain C. Sato M. Schwartz J.-L. (2014). A possible neurophysiological correlate of audiovisual binding and unbinding in speech perception. Front. Psychol. 5:1340. 10.3389/fpsyg.2014.01340 25505438 PMC4244540

[B12] Heald S. Nusbaum H. C. (2014). Talker variability in audiovisual speech perception. Front. Psychol. 5:698. 10.3389/fpsyg.2014.00698 25076919 PMC4100456

[B13] Irwin J. Brancazio L. (2014). Seeing to hear? Patterns of gaze to speaking faces in children with autism spectrum disorders. Front. Psychol. 5:397. 10.3389/fpsyg.2014.00397 24847297 PMC4021198

[B14] Ito T. Gracco V. L. Ostry D. J. (2014). Temporal factors affecting somatosensory-auditory interactions in speech processing. Front. Psychol. 5:1198. 10.3389/fpsyg.2014.01198 25452733 PMC4233986

[B15] Jenson D. Bowers A. L. Harkrider A. Thornton D. Cuellar M. Saltuklaroglu T. (2014). Temporal dynamics of sensorimotor integration in speech perception and production: independent component analysis of EEG data. Front. Psychol. 5:656. 10.3389/fpsyg.2014.00656 25071633 PMC4091311

[B16] Kelly S. Hirata Y. Manansala M. Huang J. (2014). Exploring the role of hand gestures in learning novel phoneme contrasts and vocabulary in a second language. Front. Psychol. 5:673. 10.3389/fpsyg.2014.00673 25071646 PMC4077026

[B17] Klucharev V. Möttönen R. Sams M. (2003). Electrophysiological indicators of phonetic and non-phonetic multisensory interactions during audiovisual speech perception. Brain Res. Cogn. Brain Res. 18, 65–75. 10.1016/j.cogbrainres.2003.09.004 14659498

[B18] Lee H. L. Noppeney U. (2014). Music expertise shapes audiovisual temporal integration windows for speech, sinewave speech and music. Front. Psychol. 5:868. 10.3389/fpsyg.2014.00868 25147539 PMC4124486

[B19] Leybaert J. Macchi L. Huyse A. Champoux F. Bayard C. Colin C.. (2014). Atypical audio-visual speech perception and McGurk effects in children with specific language impairment. Front. Psychol. 5:422. 10.3389/fpsyg.2014.00422 24904454 PMC4033223

[B20] McGurk H. MacDonald J. (1976). Hearing lips and seeing voices. Nature, 264, 746–748. 10.1038/264746a0 1012311

[B21] Meronen A. Tiippana K. Westerholm J. Ahonen T. (2013). Audiovisual speech perception in children with developmental language disorder in degraded listening conditions. J. Speech Lang. Hear. Res. 56, 211–221. 10.1044/1092-4388(2012/11-0270) 22653918

[B22] Molholm S. Ritter W. Murray M. M. Javitt D. C. Schroeder C. E. Foxe J. J. (2002). Multisensory auditory-visual interactions during early sensory processing in humans: a high-density electrical mapping study. Cognitive Brain Research, 14, 115–128. 10.1016/S0926-6410(02)00066-6 12063135

[B23] Rogers J. C. Möttönen R. Boyles R. Watkins K. E. (2014). Discrimination of speech and non-speech sounds following theta-burst stimulation of the motor cortex. Front. Psychol. 5:754. 10.3389/fpsyg.2014.00754 25076928 PMC4097947

[B24] Saalasti S. Kätsyri J. Tiippana K. Laine-Hernandez M. von Wendt L. Sams M. (2012). Audiovisual speech perception and eye gaze behavior of adults with Asperger Syndrome. J. Autism Dev. Disord. 42, 1606–1615. 10.1007/s10803-011-1400-0 22068821

[B25] Scarbel L. Beautemps D. Schwartz J.-L. Sato M. (2014). The shadow of a doubt ? Evidence for perceptuo-motor linkage during auditory and audiovisual close shadowing. Front. Psychol. 5:568. 10.3389/fpsyg.2014.00568 25009512 PMC4068292

[B26] Sekiyama K. Soshi T. Sakamoto S. (2014). Enhanced audiovisual integration with aging in speech perception: a heightened McGurk effect in older adults. Front. Psychol. 5:323. 10.3389/fpsyg.2014.00323 24782815 PMC3995044

[B27] Shams L. Seitz A. R. (2008). Benefits of multisensory learning. Trends Cogn. Sci. 12, 411–417. 10.1016/j.tics.2008.07.006 18805039

[B28] Stevenson R. A. Segers M. Ferber S. Barense M. D. Wallace M. T. (2014). The impact of multisensory integration deficits on speech perception in children with autism spectrum disorders. Front. Psychol. 5:379. 10.3389/fpsyg.2014.00379 24904448 PMC4033130

[B29] Tiippana K. (2014). What is the McGurk effect? Front. Psychol. 5:725. 10.3389/fpsyg.2014.00725 25071686 PMC4091305

[B30] Treille A. Vilain C. Sato M. (2014). The sound of your lips: electrophysiological cross-modal interactions during hand-to-face and face-to-face speech perception. Front. Psychol. 5:420. 10.3389/fpsyg.2014.00420 24860533 PMC4026678

